# Comparative analysis of the safety and effectiveness of Nirmatrelvir-Ritonavir and Azvudine in older patients with COVID-19: a retrospective study from a tertiary hospital in China

**DOI:** 10.3389/fphar.2024.1362345

**Published:** 2024-07-22

**Authors:** Nan Shang, Xianlin Li, Zhiyu Guo, Lan Zhang, Shanshan Wang

**Affiliations:** ^1^ Department of Pharmacy, The First Hospital of Shanxi Medical University, Taiyuan, Shanxi, China; ^2^ School of Pharmacy, Shanxi Medical University, Taiyuan, Shanxi, China; ^3^ School of Public Health, Capital Medical University, Beijing, China; ^4^ Section of Occupational Medicine, Department of Special Medicine, Shanxi Medical University, Taiyuan, China

**Keywords:** COVID-19, Nirmatrelvir-Ritonavir, Azvudine, effectiveness, safety

## Abstract

**Introduction:** Numerous studies have explored the treatment outcomes of Nirmatrelvir-Ritonavir and Azvudine in older patients with COVID-19. However, direct comparisons between these two drugs are still relatively limited. This study aims to compare the safety and effectiveness of these two drugs in Chinese older patients with early infection to provide strategies for clinical treatment.

**Methods:** Older COVID-19 patients (age ≥65) hospitalized during the winter 2022 epidemic in China were included and divided into Nirmatrelvir-Ritonavir and Azvudine. Demographics, medication information, laboratory parameters, and treatment outcomes were collected. All-cause 28-day mortality, delta cycle threshold (ΔCt), nucleic acid negative conversion time, and incidence of adverse events were defined as outcomes. Propensity score matching (PSM), Kaplan-Meier, Cox proportional hazards model, subgroup analysis, and nomograms were selected to evaluate the outcomes.

**Results:** A total of 1,508 older COVID-19 patients were screened. Based on the inclusion and exclusion criteria, 1,075 patients were eligible for the study. After PSM, the final number of older COVID-19 patients included in the study was 375, and there were no significant differences in demographic characteristics between the two groups (*p* > 0.05). Compared to the Azvudine group, the Nirmatrelvir-Ritonavir group showed a higher incidence of multiple adverse events (12.8% vs 5.2%, *p* = 0.009). The incidence of adverse events related to abnormal renal function was higher in the Nirmatrelvir-Ritonavir group compared to the Azvudine group (13.6% vs 7.2%, *p* = 0.045). There were no significant differences between the two groups in terms of all-cause 28-day mortality (HR = 1.020, 95% CI: 0.542 - 1.921, *p* = 0.951), whereas there were significant differences in nucleic acid negative conversion time (HR = 1.659, 95% CI: 1.166 - 2.360, *p* = 0.005) and ΔCt values (HR = 1.442, 95% CI: 1.084 - 1.918, *p* = 0.012).

**Conclusion:** Azvudine and Nirmatrelvir-Ritonavir have comparable effectiveness in reducing mortality risk. Azvudine may perform better in nucleic acid negative conversion time and virus clearance and shows slightly better safety in older patients. Further studies with a larger sample size were needed to validate the result.

## 1 Background

COVID-19, caused by the SARS-CoV-2 virus, has significantly impacted global health, especially among the older population due to their age-related decline in immune function and a higher prevalence of comorbidities (Bartleson et al., 2021). Older individuals were particularly susceptible to severe outcomes from COVID-19 due to their weakened immune response and higher likelihood of having underlying health conditions that exacerbate the severity of the disease (Sanyaolu et al., 2020; Bartleson et al., 2021). Epidemiological data have revealed that individuals aged 65 years and over accounted for more than 60% of hospital admissions and a considerable proportion of ICU admissions in Northern Italy, indicating higher hospitalization and mortality rates in this demographic (Azzolina et al., 2022). Notably, a mortality rate as high as 30% has been observed in patients aged 80 and above, in stark contrast to less than 5% for those below 60 (Bonanad et al., 2020). Among these patients, prolonged hospital stays and an increased need for ventilatory support have been frequently observed, along with a higher incidence of post-hospitalization complications (O'Mahoney et al., 2023). Additionally, the presence of comorbidities such as hypertension, diabetes, and chronic respiratory diseases significantly exacerbated the risk of severe outcomes (Sanyaolu et al., 2020). Therefore, the development and evaluation of COVID-19 treatments have been necessitated to specifically include older patients, ensuring the representativeness of data for this particular group. Therefore, in the development and evaluation of COVID-19 treatments, older patients must be specifically included to ensure the research findings represent this important demographic.

In 2022, China experienced a significant breakthrough in COVID-19 treatment. This progress was marked by the National Medical Products Administration (NMPA) and the National Healthcare Security Administration (NHSA) approving the clinical use of two oral medications, Nirmatrelvir-Ritonavir and Azvudine (Zhu, 2023). These agents, which were included in the medical reimbursement list, were extensively utilized during the winter 2022 outbreak. Nirmatrelvir was formulated with Ritonavir, a pharmacokinetic enhancer. It targeted SARS-CoV-2’s main protease, known as Mpro or 3CLpro (Hashemian et al., 2023). This action was key in blocking the virus’s ability to process polyprotein precursors, thus hindering viral replication (V'Kovski et al., 2021). The combination’s enhanced efficacy was due to Ritonavir inhibiting the metabolism of Nirmatrelvir through CYP3A, increasing its plasma levels (Dian et al., 2023). In February 2022, Nirmatrelvir-Ritonavir was approved by the NMPA as the first treatment for mild to moderate COVID-19 cases in China primarily for adult patients (Shi et al., 2023). Azvudine, identified as a synthetic nucleoside analog, was recognized for its effects exerted through phosphorylation within cells, leading to the formation of its active metabolite, Azvudine triphosphate. It received conditional authorization from the NMPA for treating COVID-19 in China on 25 July 2022 (Zhu, 2023).

Previous studies have provided empirical data on the use of Nirmatrelvir-Ritonavir and Azvudine in the treatment of COVID-19, especially in older populations (Wee et al., 2023). Nirmatrelvir-Ritonavir showed effectiveness in reducing the disease’s severity and mortality (Talic et al., 2021; Nyberg et al., 2022; Hughes et al., 2023; Jing et al., 2023; Najjar-Debbiny et al., 2023). Azvudine has also shown generally good effectiveness and safety in early stages of infection, but more real-world multi-center data were still needed (Chen and Tian, 2023b). Other options besides these two drugs also exist, and have demonstrated potential therapeutic value in early infection (Mazzitelli et al., 2023a; Mazzitelli et al., 2023b; Grundeis et al., 2023; Kip et al., 2023; Wong et al., 2023). One such drug, Remdesivir, works by inhibiting COVID-19 replication by interfering with viral RNA synthesis (Grundeis et al., 2023). Monoclonal antibody treatment neutralizes the virus by targeting surface proteins, thereby reducing the severity of symptoms caused by infection (Kip et al., 2023; Lin et al., 2023). Similarly, Molnupiravir disrupts viral replication by introducing errors into the viral genome (Teli et al., 2023; Mazzitelli et al., 2023a; Wong et al., 2023; Mazzitelli et al., 2023b). Although these pharmacological interventions expand the scope of treatment, additional studies are needed to characterize their safety in older groups. Furthermore, due to regulatory approval, Nirmatrelvir-Ritonavir and Azvudine have achieved wider adoption in clinical settings in China than alternative medicines.

The principal objective of this study was to compare the safety and effectiveness of Nirmatrelvir-Ritonavir and Azvudine in the treatment of older patients with early infection of COVID-19. The differences in treatments, outcomes, and adverse effects of the two medications were specifically assessed and analyzed in this study. This comparison would provide valuable insight into the optimal management of early infection of COVID-19 in older patients, a demographic that remains at high risk of severe disease and complications.

## 2 Methods

### 2.1 Study design and settings

This retrospective cohort study was conducted at the First Hospital of Shanxi Medical University, a premier tertiary hospital in Shanxi Province. The study was spanned from December 2022 to February 2023, a period that coincided with a major COVID-19 outbreak in China. The substantial medical capabilities of the hospital and its history of managing a large influx of COVID-19 patients since the lockdown period rendered it an ideal site for the study. Furthermore, the use of both therapeutic medications, Nirmatrelvir-Ritonavir and Azvudine, at the hospital was a crucial factor in its selection as the research setting. This study was approved by the Ethics Committee of the First Hospital of Shanxi Medical University (Ethics Number: NO. KYLL-2023-089, Approval Date: 27 March 2023).

### 2.2 Participants

Patients aged 65 and older, diagnosed with COVID-19 and hospitalized during the study period, were the primary subjects of the study. They were identified from the hospital’s electronic medical records (EMRs). Inclusion criteria included patients 1) have a confirmed COVID-19 diagnosis via a reverse transcriptase polymerase chain reaction (RT-PCR); 2) being 65 years or older; and 3) receiving treatment with either Nirmatrelvir-Ritonavir or Azvudine during early infection. Early infection of COVID-19 was defined as within 14 days from the onset of symptoms to the initiation of antiviral therapy. Exclusion criteria were patients 1) under 65 years or not having undergone treatment with Nirmatrelvir-Ritonavir or Azvudine during early infection; 2) a history of allergic reactions to either of these medications; 3) having incomplete clinical records, either laboratory test results or drug usage information while hospitalized; and 4) not belong to early infection.

### 2.3 Data collection

Patient data for this study were extracted from the EMRs and included patient demographics (age, gender, BMI, smoking and drinking habits, the severity of COVID-19, comorbidities such as diabetes, cardiovascular diseases, hypertension), treatment regimen (time from hospitalization to initiation of antiviral therapy, duration of medication treatment, number of prescribed doses), laboratory results (liver function, renal function, coagulation, inflammation tests, others), and treatment outcomes (all-cause 28-day mortality, ΔCt values, and nucleic acid negative conversion time). To ensure patient confidentiality, all data were anonymized. The follow-up period for the included patients in this study ended 28 days from the date of hospitalization. Patients were censored on the day of discharge if they were discharged from the hospital within 28 days. Patients who died within 28 days of hospitalization were censored on the date of death.

### 2.4 Variable definitions

COVID-19 diagnosis and severity were determined based on positive SARS-CoV-2 nucleic acid testing using RT-PCR (Seymen et al., 2023). A habit of smoking was defined as consistently smoking at least 10 cigarettes a day for more than 1 year, and a habit of drinking was defined as consistently drinking any alcohol at least once a week for more than 1 year. The severity of COVID-19 was categorized into mild, moderate, and severe categories based on the clinical assessments documented by physicians in the medical records system. Early infection was defined as the time interval from the onset of symptoms to the initiation of antiviral treatment, ensuring it does not exceed 14 days. Adverse events were assessed based on changes to biochemical parameters compared to baseline laboratory values. The nucleic acid negative conversion time is defined as whether the RT-PCR test result turned negative within 14 days while hospitalized. The ΔCt value, defined as the difference in cycle threshold between two samples during hospitalization, was classified as whether ≥3 within 14 days (Juanola-Falgarona et al., 2022; Wong et al., 2023). The severity of adverse reactions was evaluated using the Common Terminology Criteria for Adverse Events (CTCAE) version 5.0, categorizing them into mild, moderate, and severe.

### 2.5 Outcomes

The all-cause 28-day mortality was considered as the primary outcome, defined as a binary variable (death defined as 1, survival defined as 0). Secondary outcomes included the binary variables for ΔCt values (ΔCt ≥ 3 defined as 1, ΔCt < 3 defined as 0) and nucleic acid negative conversion time (negative within 14 days defined as 1, not negative within 14 days defined as 0). The all-cause 28-day mortality, ΔCt values, and nucleic acid negative conversion time were presented as proportions. Additionally, detailed records of adverse reactions pertaining to Nirmatrelvir-Ritonavir and Azvudine treatment were recorded, which were presented as frequencies (percentages).

### 2.6 Statistical analysis

Comparative analyses of baseline characteristics, safety, and effectiveness outcomes between the two medications were conducted. To control for confounding factors, propensity score matching (PSM) was employed using a “logit” model and the “nearest” method. This approach matched patients in a 1:2 ratio with a caliper value of 0.1. Age, gender, BMI, smoking and drinking habits, severity of COVID-19, and comorbidity with diabetes, cardiovascular diseases, and hypertension were selected to construct the propensity score. In the comparison analysis between the two groups, the independent samples *t*-test was used for normally distributed quantitative data, while the Mann-Whitney U test for non-normally distributed data. Categorical variables were presented as frequencies (percentages), and comparisons between groups were conducted using the chi-square test.

Kaplan-Meier method and log-rank test were selected to compare the event curves. A Cox proportional hazards model was constructed to analyze the relationship between Nirmatrelvir-Ritonavir or Azvudine groups and outcomes (all-cause 28-day mortality, nucleic acid negative conversion time, ∆Ct). Model one is the unadjusted model. Model two was adjusted for severity of COVID-19, comorbidity with cardiovascular diseases, number of prescribed doses, duration of medication treatment, and time from hospitalization to initiation of antiviral therapy. Subgroup analyses were conducted to assess the effect of the two treatments in different situations. We divided the study cohorts into subgroups of 1) mild-to-moderate COVID cases and severe COVID cases; 2) patients with cardiovascular diseases and without cardiovascular diseases; 3) duration of treatment under 5 days, 5-10 days and above 10 days; 4) time from hospitalisation to initiation of antiviral therapy under or equal to 5 days and over 5 days; 5) number of prescribed doses under 20 and above 20. Additionally, we utilized three nomograms to predict the all-cause 28-day mortality, nucleic acid negative conversion time, and the ΔCt ≥ 3 among COVID-19 patients under different treatment conditions (Wang et al., 2024).

All significance tests were double-tailed, with *p* < 0.05 considered statistically significant. All statistical analyses were performed using IBM SPSS Statistics 25 and R software (version 4.1.2).

### 2.7 Data quality control

In this study, several quality control measures were implemented to ensure the accuracy of medication exposure and outcomes. Data collection followed a clear protocol and was conducted by qualified personnel. A comprehensive data dictionary was used to define all data fields, ensuring consistency. All key data were double-entered and reviewed by independent researchers. We cross-verified the EMR data with multiple sources, including prescription review records and laboratory results. Regular data quality assessments were conducted to identify and correct errors. All data handling adhered to strict data protection and privacy regulations, ensuring the accuracy and reliability of our findings.

## 3 Results

### 3.1 Baseline characteristics of patients

**FIGURE 1:**
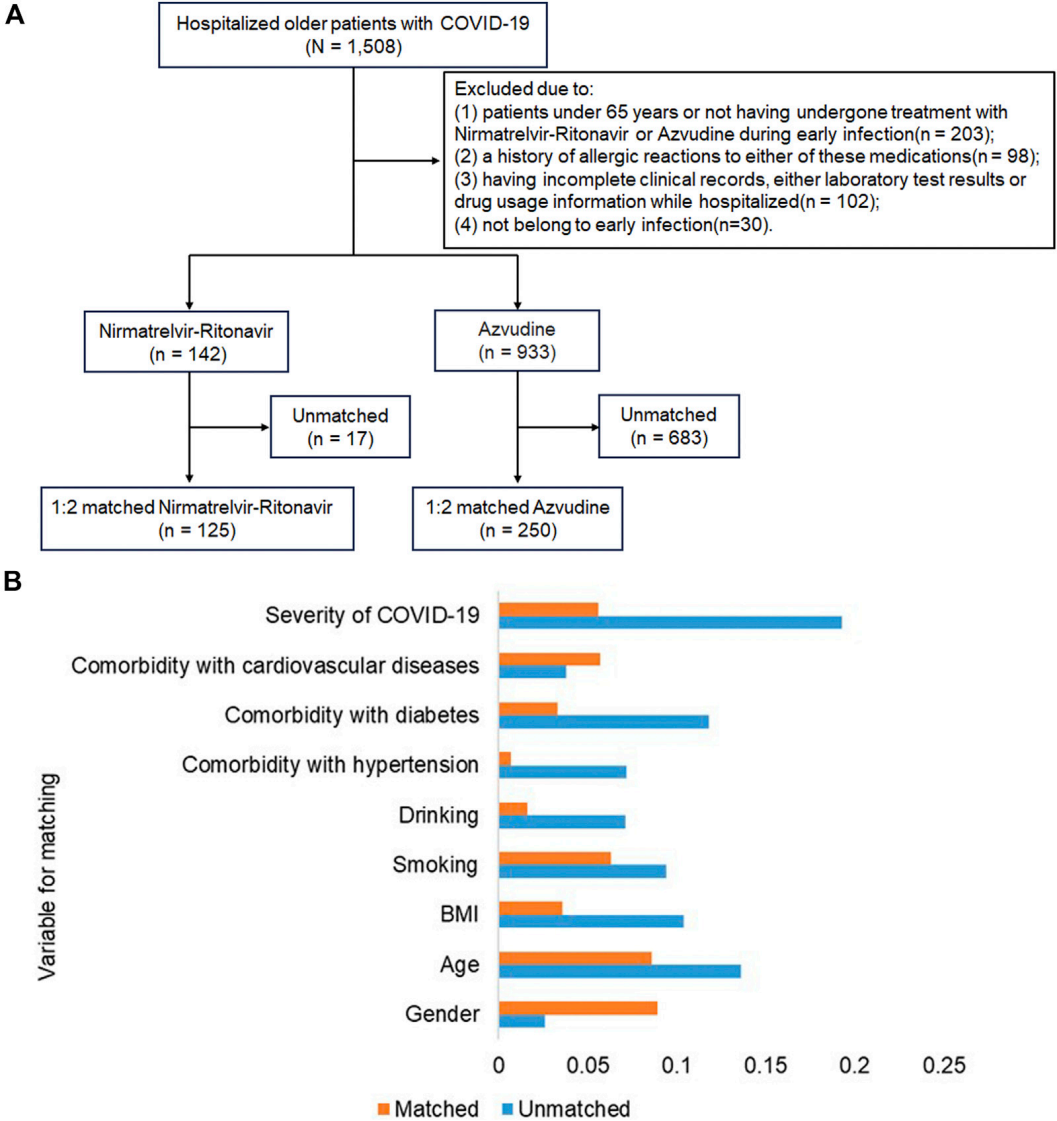
Inclusion flowchart of study subjects and standardized mean difference (SMD) between the two groups. **(A)** Flowchart demonstrating the inclusion and exclusion of older COVID-19 patients during the study period; **(B)** Standardized mean differences between the two groups before and after 1:2 propensity score matching.

From December 2022 to February 2023, a total of 1508 COVID-19 patients aged 65 years and above were screened. From these, 1075 patients were eligible for the study based on the inclusion and exclusion criteria. After propensity score matching, a final cohort of 142 patients in the Nirmatrelvir-Ritonavir group and 933 patients in the Azvudine group was selected. A final comparison was made between 125 patients treated with Nirmatrelvir-Ritonavir and 250 patients treated with Azvudine (Figure 1A). Before matching, the balance of most baseline characteristics between the two groups was sub-optimal (SMD > 0.1). However, factors such as age, gender, smoking and drinking habits, severity of COVID-19, and presence of comorbidities showed no significant differences between the two groups (Table 1). After matching, baseline characteristics of patients in both groups achieved balance, with all covariates having SMD < 0.1 (Figure 1B).

**TABLE 1:** Distribution and comparisons of baseline characteristics before and after PSM.

Baseline characteristics	Before matching patients			After 1:2 propensity-score matching patients		
	Nirmatrelvir-ritonavir (n = 142)	Azvudine (n = 933)	*p*-value	Nirmatrelvir-ritonavir (n = 125)	Azvudine (n = 250)	*p*-value
**Age, mean ± SD, year**	79.98 ± 8.82	79.01 ± 7.60	0.361	79.94 ± 8.86	78.80 ± 7.81	0.207
**BMI, mean ± SD**	24.02 ± 4.35	24.25 ± 6.02	0.249	23.23 ± 4.40	24.02 ± 9.90	0.394
**Gender, n (%)**			0.145			0.816
Male	98 (69.0)	585 (62.7)		83 (66.4)	169 (67.6)	
Female	44 (31.0)	348 (37.3)		42 (33.6)	81 (32.4)	
**Smoking, n (%)**	47 (33.1)	410 (43.9)	0.015	32 (25.6)	54 (21.6)	0.385
**Drinking, n (%)**	22 (15.5)	355 (38.0)	<0.001	15 (12.0)	36 (14.4)	0.523
**Severity of COVID-19, n (%)**			0.131			0.080
Mild-to-moderate	72 (50.7)	536 (57.4)		55 (44.0)	134 (53.6)	
Severe	70 (49.3)	397 (42.6)		70 (56.0)	116 (46.4)	
**Comorbidity with diabetes, n (%)**	50 (35.2)	300 (32.2)	0.469	46 (36.8)	78 (31.2)	0.277
**Comorbidity with hypertension, n (%)**	72 (50.7)	479 (51.3)	0.888	68 (54.4)	127 (50.8)	0.511
**Comorbidity with cardiovascular diseases, n (%)**	37 (26.1)	258 (27.7)	0.691	28 (22.4)	60 (24.0)	0.730

After propensity score matching, a final cohort of 142 patients in the Nirmatrelvir-Ritonavir group and 933 patients in the Azvudine group was selected.

### 3.2 Treatment regimen

After PSM, a comparison was made on the treatment regimen between both Nirmatrelvir-Ritonavir and Azvudine groups, including time from hospitalization to initiation of Nirmatrelvir-Ritonavir or Azvudine therapy, duration of medication treatment, and number of prescribed doses. The findings showed significant differences in the duration of medication treatment between the two groups (*p* < 0.001), with 5–10 days being the most common duration for both groups. There were no significant differences in the timing of treatment initiation (*p* = 0.189), or the number of prescribed doses (*p* = 0.307) between the two groups. (Table 2).

**TABLE 2:** Comparison of medication prescription information between the two groups.

Medication information	Nirmatrelvir-ritonavir (n = 125)	Azvudine (n = 250)	*χ* ^*2*^	*p*-value
**Duration of medication treatment, n (%)**			25.822	<0.001
0-5	54 (43.2)	52 (20.8)		
5–10	57 (45.6)	128 (51.2)		
>10	14 (11.2)	70 (28.0)		
**Time from hospitalization to initiation of antiviral therapy, n (%)**			1.726	0.189
≤5	102 (81.6)	189 (75.6)		
>5	23 (18.4)	61 (24.4)		
**Number of prescribed doses, n (%)**			1.045	0.307
0–20	58 (46.4)	130 (52.0)		
>20	67 (53.6)	120 (48.0)		

### 3.3 Safety outcomes

The safety assessment revealed a slightly higher incidence of adverse events in the Nirmatrelvir-Ritonavir group compared to Azvudine (16% vs 13.2%, *p* = 0.463). Specifically, the proportions with more than one adverse event (12.8% vs 5.2%, *p* = 0.009) and abnormal renal function (13.6% vs 7.2%, *p* = 0.045) appeared to be higher in the Nirmatrelvir-Ritonavir group compared to the Azvudine group (Table 3).

**TABLE 3:** Comparison of the safety of two medications in the treatment of older patients with COVID-19.

Adverse event category	Nirmatrelvir-ritonavir (n = 125)		Azvudine (n = 250)		*p*-value
	n	%	n	%	
Any adverse event	20	16.0	33	13.2	0.463
More than one adverse event	16	12.8	13	5.2	0.009
Severity					0.414
Mild	34	27.2	62	24.8	
Moderate	7	5.6	18	7.2	
Severe	3	2.4	15	6.0	
Abnormal liver function	2	1.6	8	3.2	0.365
Abnormal renal function	17	13.6	18	7.2	0.045
Inflammation	20	16.0	33	13.2	0.463
Abnormal coagulation function	51	40.8	113	45.2	0.418
Other	5	4.0	3	1.2	0.165

### 3.4 Effectiveness outcomes

A comparison of the effectiveness between the Nirmatrelvir-Ritonavir and Azvudine groups was conducted (Table 4). The primary outcome measure was the all-cause 28-day mortality, while secondary outcome measures included nucleic acid negative conversion time, and ΔCt values.

**TABLE 4:** Comparison of the effectiveness of two medications in the treatment of older patients with COVID-19.

Treatment outcomes	Nirmatrelvir-ritonavir	Azvudine	χ^2^	*p*-value
Primary Outcome				
**All-cause 28-day mortality**	(N = 117)	(N = 240)	0.131	0.717
No	103 (88.0)	208 (86.7)		
Yes	14 (12.0)	32 (13.3)		
Secondary Outcome				
**Nucleic acid negative conversion time**	(N = 115)	(N = 235)	6.112	0.013
No	70 (60.9)	110 (46.8)		
Yes	45 (39.1)	125 (53.2)		
**ΔCt value**	(N = 115)	(N = 235)	6.896	0.009
No	44 (38.3)	58 (24.7)		
Yes	71 (61.7)	177 (75.3)		

In terms of all-cause 28-day mortality, the mortality rate in the Nirmatrelvir-Ritonavir group was lower than in the Azvudine group (12.0% vs 13.3%, *p* = 0.717). Significant differences were observed between the two groups in terms of nucleic acid negative conversion time and ΔCt values. The nucleic acid negative conversion rate was significantly higher in the Azvudine group compared to the Nirmatrelvir-Ritonavir group (53.2% vs 39.1%, *p* = 0.013). Additionally, a significantly higher proportion of patients in the Azvudine group achieved ΔCt values compared to the Nirmatrelvir-Ritonavir group (75.3% vs 61.7%, *p* = 0.009).

**FIGURE 2:**
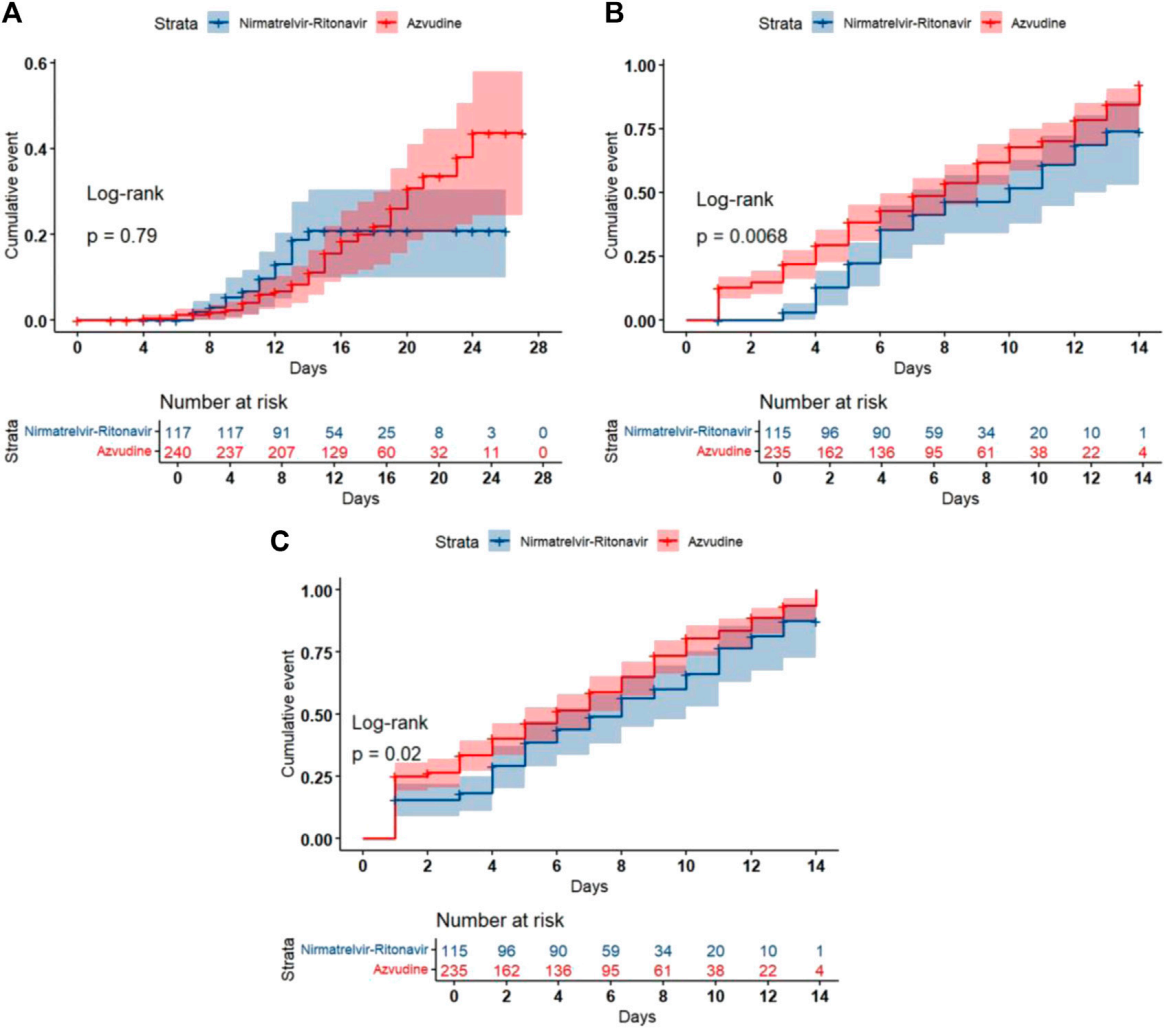
Kaplan-Meier curves of the clinical outcomes. **(A)** All-cause 28-day mortality; **(B)** Nucleic acid negative conversion time; **(C)** ΔCt values.

Kaplan-Meier method and log-rank test were used to compare the outcome events of different antiviral intervention groups. The all-cause 28-day mortality did not differ significantly between the two groups (Log-rank *p* = 0.79) (Figure Figure2A). The Azvudine group exhibited significantly faster nucleic acid negative conversion time compared to the Nirmatrelvir-Ritonavir group (Log-rank *p* = 0.0068) (Figure 2B), and also achieved a significant advantage over the Nirmatrelvir-Ritonavir group in ΔCt ≥3 (Log-rank *p* = 0.02) (Figure 2C).

The univariate Cox proportional hazards regression analysis indicated no significant difference in all-cause 28-day mortality between Azvudine and Nirmatrelvir-Ritonavir (HR = 0.917, 95% CI: 0.489-1.722, *p* = 0.788), and the multi-adjusted Cox proportional hazard model showed that there was no significant correlation between the exposure to Nirmatrelvir-Ritonavir or Azvudine and all-cause 28-day mortality. (HR = 1.020, 95% CI: 0.542-1.921, *p* = 0.951). Additionally, compared to the Nirmatrelvir-Ritonavir group, the Azvudine group significantly reduced the nucleic acid negative conversion time (HR = 1.558, 95% CI: 1.107-2.192, *p* = 0.011). This result remained significant after adjusting for severity of COVID-19, duration of medication treatment, time from hospitalization to initiation of antiviral therapy, and number of prescribed doses (HR = 1.659, 95% CI: 1.166 - 2.360, *p* = 0.005). Furthermore, compared to the Nirmatrelvir-Ritonavir group, the Azvudine group significantly increased ΔCt values (HR = 1.346, 95% CI: 1.021 - 1.773, *p* = 0.035). This result also remained significant after adjusting for severity of COVID-19, duration of medication treatment, time from hospitalization to initiation of antiviral therapy, and number of prescribed doses (HR = 1.442, 95% CI: 1.084 - 1.918, *p* = 0.012) (see Table 5 and Supplementary materials)

**TABLE 5:** Cox proportional hazards regression analysis of different antiviral treatment and clinical outcomes.

Group	Model1		Mode2	
	Crude HR (95% CI)	*p*-value	Adjusted HR (95%CI)	*p*-value
All-cause 28-day mortality				
Naimatevir-Ritonavir	References		References	
Azvudine	0.917 (0.489–1.722)	0.788	1.020 (0.542–1.921)	0.951
Nucleic acid negative conversion time				
Naimatevir-Ritonavir	References		References	
Azvudine	1.558 (1.107–2.192)	0.011	1.659 (1.166–2.360)	0.005
ΔCt value				
Naimatevir-Ritonavir	References		References	
Azvudine	1.346 (1.021–1.773)	0.035	1.442 (1.084–1.918)	0.012

Model 1: Crude model. Model 2: All-cause 28-day mortality was adjusted for severity of COVID-19, comorbidity with cardiovascular diseases, and number of prescribed doses; Nucleic acid negative conversion time and ΔCt, value difference were adjusted for severity of COVID-19, duration of medication treatment, time from hospitalization to initiation of antiviral therapy, and number of prescribed doses. Abbreviations: HR, hazard ratio; 95% CI, 95% Confidence Interval.

### 3.5 Subgroup analysis and nomogram model

**FIGURE 3:**
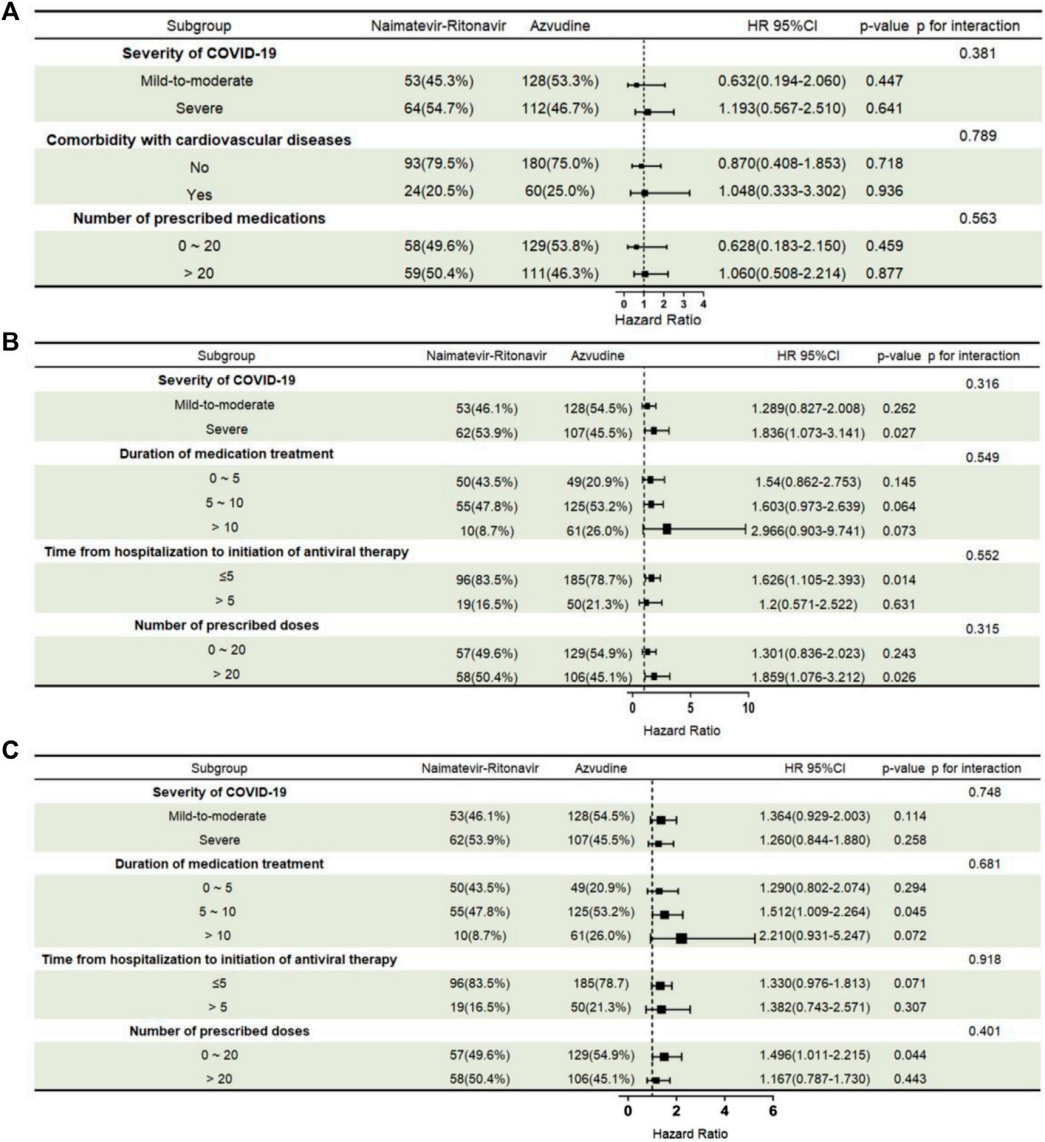
Subgroup analysis of different antiviral treatments: all-cause 28-day mortality, nucleic acid negative conversion time, and ΔCt values. **(A)** All-cause 28-day mortality; **(B)** Nucleic acid negative conversion time; **(C)** ΔCt values. Subgroups and subgroup-treatment interactions were added to the model to evaluate differences in treatment effects between subgroup categories. Abbreviations: HR, hazard ratio; 95% CI, 95% confidence interval.

In subgroup analysis, there was no significant difference in all-cause 28-day mortality between Azvudine and Nirmatrelvir-Ritonavir under different conditions of severity of COVID-19, comorbidity with cardiovascular diseases, and number of prescribed doses (Figure 3A). In the subgroup analyses for the nucleic acid negative conversion time, Azvudine and Nirmatrelvir - Ritonavir showed significant differences in their effects on the conversion time in the subgroups with severe COVID-19 (HR = 1.836, 95% CI: 1.073 - 3.141, *p* = 0.027), time from hospitalization to initiation of antiviral therapy under 5days (HR = 1.626, 95% CI: 1.105 - 2.393, *p* = 0.014), and number of prescribed doses over 20 (HR = 1.859, 95% CI: 1.076 - 3.212, *p* = 0.026) (Figure 3B). In the subgroup analyses for the ΔCt values, Azvudine and Nirmatrelvir - Ritonavir showed significant differences in their effects on the increased values in the subgroups with duration of medication treatment of 5–10 days (HR = 1.512, 95% CI: 1.009 - 2.264, *p* = 0.045) and a prescribed dose range of 0–20 (HR = 1.496, 95% CI: 1.011 - 2.215, *p* = 0.044) (Figure 3C).

**FIGURE 4:**
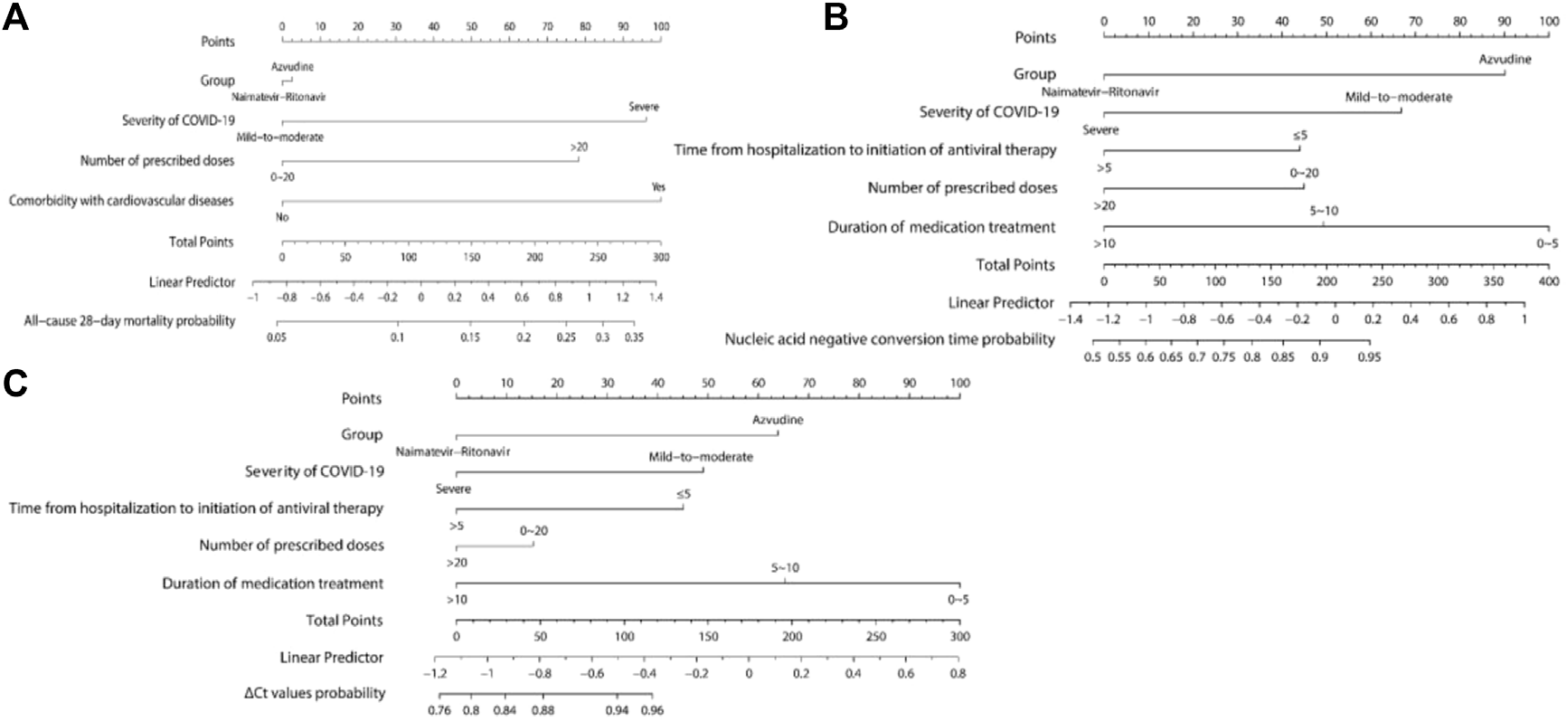
Nomograms Predicting all-cause 28-day mortality, nucleic acid negative conversion time, and ΔCt values. **(A)** All-cause 28-day mortality; **(B)** Nucleic acid negative conversion time; **(C)** ΔCt values.

Three nomograms were developed: the first one, constructed according to group, comorbidity with cardiovascular diseases, the severity of COVID-19, and the number of prescribed doses (Figure 4A), is used to predict all-cause 28-day mortality; the second (Figure 4B) and third (Figure 4C) nomograms, constructed based on group, severity of COVID-19, duration of medication treatment, time from hospitalization to initiation of antiviral therapy, and number of prescribed doses, are used to predict nucleic acid negative conversion time and ΔCt values, respectively. As shown in the nomograms, each variable corresponds to a specific score. By summing the variable scores and mapping them to the total score, the predicted rates of all-cause 28-day mortality, nucleic acid negative conversion, and ΔCt values for the two drugs can be determined.

## 4 Discussion

This study compared the safety and effectiveness of Nirmatrelvir-Ritonavir and Azvudine in older patients hospitalized with COVID-19. A cohort of 375 subjects was selected from a pool of patients at a tertiary hospital in China during the peak of the pandemic. During that time, both Nirmatrelvir-Ritonavir and Azvudine had already been approved for clinical use and were widely available. Our main findings indicate that there is no significant difference between Azvudine and Nirmatrelvir-Ritonavir in reducing the risk of mortality. However, Azvudine may perform better in reducing nucleic acid conversion time and lowering viral load, as well as demonstrating slightly better safety in older patients.

The results indicate that, in the Nirmatrelvir-Ritonavir group, the duration of medication administration was mainly concentrated in the 5–10 days range, whereas in the Azvudine group, it was primarily in the 5-10 days and over 10-day ranges, with a significant difference observed (*p* < 0.001) (Table 2). According to existing clinical guidelines, Nirmatrelvir-Ritonavir was typically recommended for a 5-day course, while Azvudine was recommended for a 7-day course, with potential adjustments based on the patient’s condition in actual clinical practice. The findings suggested that the use of Nirmatrelvir-Ritonavir aligned more closely with standardized treatment recommendations. In contrast, the use of Azvudine in clinical practice remained in an exploratory and adjustment phase, indicating the need for more evidence to support its optimal use. Regarding the time from hospitalization to the initiation of antiviral therapy, both groups had a similar proportion of patients starting treatment within 5 days of hospitalization. This indicated that in clinical practice, regardless of the medication used, physicians tended to initiate antiviral therapy as early as possible to achieve better therapeutic outcomes. This was consistent with existing guidelines (Bryant, 2020; COVID-19 Treatment Guidelines Panel, 2024). Besides, the broad categories for duration of medication treatment, time from hospitalization to initiation of antiviral therapy, and number of prescribed doses were chosen to reflect clinical guidelines and typical practices, ensuring consistency across the study population. However, this approach may reduce the granularity of the findings suggesting that future studies consider more detailed categorizations to enhance interpretability.

In terms of safety, it was found that following treatment with Azvudine, there was a lower incidence of abnormal renal function (13.6% vs 7.2%, *p* = 0.045), and one or more adverse events (12.8% vs 5.2%, *p* = 0.009) compared to Nirmatrelvir-Ritonavir. These results suggest that Azvudine may have lower hepatotoxicity, nephrotoxicity, and metabolic impact in certain aspects. A study on Nirmatrelvir-Ritonavir, showed that Ritonavir was primarily metabolized in the liver, while Nirmatrelvir could accumulate in the kidney and increase the incidence of abnormal renal function (Lingscheid et al., 2022). This is a concern for older patients, who, with advancing age and the presence of comorbidities such as diabetes, hypertension, and cardiovascular diseases, a reduced renal excretion rate could exacerbate the occurrence of adverse events (Kellum et al., 2021). In a previous COVID-19 study involving patients with renal failure, Azvudine showed fewer adverse reactions related to abnormal renal function compared to the standard treatment group (patients receiving standard antiviral treatment, symptomatic treatment, nutritional support, and oxygen inhalation) (Shang et al., 2023). Above all, it is important to be cautious when administering either of these two medications in older COVID-19 patients, especially those with abnormal renal function. It is important to note that all adverse events identified in this study have been previously reported in the literature and were consistent with known safety profiles of the medications, and no new or unreported adverse events were observed in our study. This consistency provides further evidence of the reliability of existing data regarding the adverse effects of these medications and their applicability to older patients with COVID-19.

This study confirms that Azvudine and Nirmatrelvir-Ritonavir have comparable effectiveness in reducing mortality risk, with Azvudine potentially showing better performance in shortening nucleic acid negative conversion time and increasing ΔCt values (Table 5). This has been confirmed by the results of a large number of previous real-world studies and clinical trials, especially in Nirmatrelvir-Ritonavir, which was the first-line treatment medication worldwide (Lewnard et al., 2023; Wong et al., 2023). According to a large cohort study in the United States of America, Nirmatrelvir-Ritonavir effectively reduces the risk of hospitalization or death within 30 days of a positive outpatient SARS-CoV-2 test (Lewnard et al., 2023). Nirmatrelvir-Ritonavir treatment also resulted in significant reductions in hospitalization and mortality from COVID-19 in patients over 65 years of age due to its rapid ability to inhibit viral replication (Al-Obaidi et al., 2023). However, due to potential interactions with other medications (such as digoxin, cyclosporine, and verapamil), careful management and monitoring are required in clinical application. These interactions can alter the metabolism and effectiveness of the antiviral drug, potentially leading to suboptimal therapeutic outcomes or increased toxicity (Stone, J. H., et al., 2020). Particularly in older patients, who often have multiple comorbidities and complex medication regimens, the risk of adverse drug interactions is higher, necessitating a thorough review of their medication history and close monitoring during treatment (Rahman et al., 2020). Moreover, our study found that Azvudine showed better performance in nucleic acid negative conversion time and virus clearance, which is particularly relevant for patients who are also on multiple medications for chronic conditions. The fewer drug interactions associated with Azvudine make it a more suitable option for these patients, reducing the risk of adverse effects and improving treatment safety.

In 2022, Azvudine became China’s first oral SARS-CoV-2RdRP inhibitor for the treatment of COVID-19 in adults. Although there are not many reported studies, multiple studies confirmed that Azvudine had a faster nucleic acid negative conversion time compared with standard antiviral treatment (Ren et al., 2020; de Souza et al., 2023; Zhao et al., 2023). A phase III multi-center randomized clinical study also showed that Azvudine could shorten the time to symptom improvement and increase the proportion of patients with mild to moderate COVID-19 (Yu and Chang, 2022). In our study, it was found that Azvudine demonstrated better performance in the time for nucleic acid negative conversion time (HR = 1.659, 95% CI: 1.166 - 2.360, *p* = 0.005) and ΔCt values (HR = 1.346, 95% CI: 1.021 - 1.773, *p* = 0.035) (Table 5) compared to Nirmatrelvir-Ritonavir. However, another study conducted by Xiang et al. comparing the same two drugs in patients with mild symptoms, found that Nirmatrelvir-Ritonavir had a better effect than Azvudine (Zhao et al., 2023). This difference might stem from the varying mechanisms of action and effects of the two medications in different disease conditions, patient populations, or differing stages of COVID-19 infection.

In this study, ΔCt values were also selected as one of the vital indicators for assessing seroconversion during the early stage of COVID-19 (Juanola-Falgarona et al., 2022). These values were also selected as an outcome indicator to compare the antiviral effectiveness of Azvudine and Nirmatrelvir-Ritonavir in a prior study (Wang et al., 2024). Similarly, in a retrospective observational study in Hong Kong, ΔCt values were used to observe the viral load among symptomatic COVID-19 patients (Lin et al., 2023). Therefore, in this study, ΔCt values were also considered as one of the outcome measures, aimed at comparing the viral load in the two treatment groups during early infection. The crude model’s results provide an initial assessment of the treatment effects, which might be influenced by confounders and therefore may not accurately reflect the true effects of the drugs. In contrast, the partially adjusted model, by controlling for confounding factors, presents a clearer demonstration of treatment effectiveness, thereby enhancing the robustness and reliability of our findings. This approach helps mitigate biases. Studies by Zheng et al. (Zheng et al., 2022; Wang et al., 2024) have explored the use of crude and partially adjusted models to assess the effectiveness of antiviral medications under different conditions, further supporting the importance and necessity of employing partially adjusted models in our study.

This study has several key strengths that enhance the robustness and relevance of our findings. Firstly, our study specifically focused on older adults, a demographic that is particularly vulnerable to severe outcomes from COVID-19. This focus enhances the applicability of our findings to clinical practices aimed at improving care for this high-risk group. The patient population at the First Hospital of Shanxi Medical University is diverse, reflecting various geographic and socioeconomic backgrounds from Shanxi Province and similar regions in China. Patients had a wide range of COVID-19 severity, from mild to critical cases. The study focused on older adults (age ≥65) with multiple comorbidities, such as diabetes and cardiovascular diseases. As a tertiary hospital, it had access to advanced medical resources and treatments, including Nirmatrelvir-Ritonavir and Azvudine. This enhances the generalizability of our findings to similar tertiary hospital settings. Secondly, by directly comparing the two treatments within this critical population, our study provides valuable insights that can inform clinical decision-making and optimize treatment strategies for older patients with COVID-19. Although multiple studies worldwide have confirmed Nirmatrelvir-Ritonavir’s status as the drug of choice in the first-line treatment of COVID-19 due to its effectiveness and safety profile, there are relatively fewer studies on the use of Azvudine. Moreover, where Azvudine was the focus of the study or where literature provides a real-world head-to-head comparison of the effectiveness and safety of the two drugs (Chen and Tian, 2023a; Wei et al., 2023; Zhu, 2023), there is little research of their use in older patients, a particularly vulnerable group. Compared with young and middle-aged patients, the mortality rate of older COVID-19 patients was much higher (Kang and Jung, 2020; Liu et al., 2020) and warrants more attention. PSM was employed to balance baseline characteristics, and a retrospective study approach was used for comparative analysis. The safety and effectiveness of these medications were assessed using Kaplan-Meier methods, Cox proportional hazards models, subgroup analysis, and nomograms.

## 5 Conclusion

Although the current study is based on a small sample of patients, the results indicated no significant difference between Azvudine and Nirmatrelvir-Ritonavir in terms of all-cause 28-day mortality. However, Azvudine showed a certain advantage in shortening nucleic acid negative conversion time and increasing ΔCt values. When comparing the safety of the two drugs, the incidence of adverse events was lower in patients treated with Azvudine than in those treated with Nirmatrelvir-Ritonavir.

## 6 Limitation

The small scale of the study and its focus on a specific period and region may limit the generalizability of the results to other groups of patients of a similar age. The impact of the number of combined medications on the effectiveness of Nirmatrelvir-Ritonavir and Azvudine treatment groups was analyzed, but a detailed analysis of specific medications was not performed. Additionally, the assessment of adverse events relied solely on medical records, which may not have fully captured their true incidence and severity. Furthermore, the lack of daily PCR testing, caused by the strain on medical resources during the pandemic, could introduce bias in the number of days to conversion to negative, potentially affecting the accuracy of our findings. Future research with larger sample sizes, standardized testing protocols, and more rigorously designed studies are needed to confirm and validate our findings and provide more robust evidence to support our conclusions.

## Data Availability

The raw data supporting the conclusions of this article will be made available by the authors, without undue reservation.
