# Supplementary material for: Comparative analysis of the safety and effectiveness of Nirmatrelvir-Ritonavir and Azvudine in older patients with COVID-19: a retrospective study from a tertiary hospital in China
Source: Front Pharmacol. 2024 Jul 22;15:1362345. doi: 10.3389/fphar.2024.1362345 (PMC11298358; doi:10.3389/fphar.2024.1362345)
Supplement: Supplementary file 1 [file Table1.DOCX]

**Comparative analysis of the safety and effectiveness of Nirmatrelvir-Ritonavir and Azvudine in older patients with COVID-19: a retrospective study from a tertiary hospital in China**

Nan Shang^1a*^, Xianlin Li^2a^, Zhiyu Guo^2^, Lan Zhang^3^, Shanshan Wang^4^

1. Department of Pharmacy, The First Hospital of Shanxi Medical University, Taiyuan, Shanxi, 030001, China

2. School of Pharmacy, Shanxi Medical University, Taiyuan Shanxi, 030001, China

3. School of Public Health, Capital Medical University, Beijing 100069, China

4. Section of Occupational Medicine, Department of Special Medicine, Shanxi Medical University, Taiyuan Shanxi, 030001, China

* Corresponding author: Nan Shang, PhD

E-mail: shangnan@vip.163.com

ORCID#: 0000-0003-0314-2859

Contact Cell Phone: +86-13834667671

a Co-first author.

Acknowledgment

This research was supported by the COVID-19 Research Project of Shanxi Provincial Health Commission (Grant No. 2023XG024).

**Supplementary information**

**Table of Contents**

Table S1 Univariate and multivariate Cox proportional hazards regression analysis of the proportion of all-cause 28-day mortality for main cohort.

Table S2 Univariate and multivariate Cox proportional hazards regression analysis of the proportion of nucleic acid negative conversion time for main cohort.

Table S3 Univariate and multivariate Cox proportional hazards regression analysis of the proportion of ΔCt value for main cohort.

Table S1 Univariate and multivariate Cox proportional hazards regression analysis of the proportion of all-cause 28-day mortality for main cohort.

| **Factor** | **Model 1** |  | **Model 2** |  |
| --- | --- | --- | --- | --- |
|  | **Crude HR 95% CI** | **p-**  **value** | **Adjusted HR 95% CI** | **p-value** |
| **Group** |  |  |  |  |
| Naimatevir-Ritonavir | Reference |  | Reference |  |
| Azvudine | 0.917(0.489-1.722) | 0.788 | 1.020(0.542-1.921) | 0.951 |
| **Gender** |  |  |  |  |
| Male | Reference |  |  |  |
| Female | 0.615(0.312-1.211) | 0.160 |  |  |
| **Age** | 1.032(0.996-1.069) | 0.085 |  |  |
| **BMI** | 0.947(0.881-1.019) | 0.144 |  |  |
| **Smoking** |  |  |  |  |
| No | Reference |  |  |  |
| Yes | 0.488(0.207-1.154) | 0.102 |  |  |
| **Drinking** |  |  |  |  |
| No | Reference |  |  |  |
| Yes | 0.621(0.222-1.736) | 0.363 |  |  |
| **Severity of COVID-19** |  |  |  |  |
| Mild-to-moderate | Reference |  | Reference |  |
| Severe | 2.169(1.138-4.132) | 0.019 | 2.044(1.056-3.956) | 0.034 |
| **Comorbidity with diabetes** |  |  |  |  |
| No | Reference |  |  |  |
| Yes | 1.198(0.653-2.200) | 0.559 |  |  |
| **Comorbidity with cardiovascular diseases** |  |  |  |  |
| No | Reference |  | Reference |  |
| Yes | 1.956(1.050-3.643) | 0.034 | 2.104(1.129-3.921) | 0.019 |
| **Comorbidity with hypertension** |  |  |  |  |
| No | Reference |  |  |  |
| Yes | 1.198(0.653-2.200) | 0.559 |  |  |
| **Duration of medication treatment** |  |  |  |  |
| 0 ~ 5 | Reference |  |  |  |
| 5 ~ 10 | 0.666(0.326-1.361) | 0.265 |  |  |
| > 10 | 0.714(0.338-1.508) | 0.377 |  |  |
| **Time from hospitalization to initiation of antiviral therapy** |  |  |  |  |
| ≤ 5 | Reference |  |  |  |
| > 5 | 0.881(0.463-1.678) | 0.701 |  |  |
| **Number of prescribed doses** |  |  |  |  |
| 0 ~ 20 | Reference |  | Reference |  |
| > 20 | 1.997(1.001-3.984) | 0.050 | 1.791(0.890-3.604) | 0.103 |

Model 1 is the unadjusted model.

Model 2 was adjusted for severity of COVID-19, comorbidity with cardiovascular diseases, and number of prescribed doses.

Abbreviations: HR, hazard ratio; 95% CI, 95% confidence interval; BMI, body mass index.

Table S2 Univariate and multivariate Cox proportional hazards regression analysis of the proportion of nucleic acid negative conversion time for main cohort.

| **Factor** | **Model 1** |  | **Model 2** |  |
| --- | --- | --- | --- | --- |
|  | **Crude HR 95% CI** | **p-value** | **Adjusted HR 95% CI** | **p-value** |
| **Group** |  |  |  |  |
| Naimatevir-Ritonavir | Reference |  | Reference |  |
| Azvudine | 1.558(1.107-2.192) | 0.011 | 1.659(1.166-2.360) | 0.005 |
| **Gender** |  |  |  |  |
| Male | Reference |  |  |  |
| Female | 0.758(0.544-1.055) | 0.100 |  |  |
| **Age** | 0.989(0.970-1.008) | 0.235 |  |  |
| **BMI** | 0.993(0.962-1.026) | 0.685 |  |  |
| **Smoking** |  |  |  |  |
| No | Reference |  |  |  |
| Yes | 0.759(0.525-1.098) | 0.143 |  |  |
| **Drinking** |  |  |  |  |
| No | Reference |  |  |  |
| Yes | 0.884(0.571-1.367) | 0.579 |  |  |
| **Severity of COVID-19** |  |  |  |  |
| Mild-to-moderate | Reference |  | Reference |  |
| Severe | 0.588(0.432-0.800) | 0.001 | 0.695(0.504-0.958) | 0.026 |
| **Comorbidity with diabetes** |  |  |  |  |
| No | Reference |  |  |  |
| Yes | 0.977(0.706-1.352) | 0.888 |  |  |
| **Comorbidity with cardiovascular diseases** |  |  |  |  |
| No | Reference |  |  |  |
| Yes | 1.30(0.924-1.835) | 0.131 |  |  |
| **Comorbidity with hypertension** |  |  |  |  |
| No | Reference |  |  |  |
| Yes | 0.861(0.637-1.164) | 0.330 |  |  |
| **Duration of medication treatment** |  |  |  |  |
| 0 ~ 5 | Reference |  | Reference |  |
| 5 ~ 10 | 0.844(0.592-1.203) | 0.348 | 0.761(0.529-1.093) | 0.139 |
| > 10 | 0.630(0.408-0.972) | 0.037 | 0.583(0.373-0.911) | 0.018 |
| **Time from hospitalization to initiation of antiviral therapy** |  |  |  |  |
| ≤ 5 | Reference |  | Reference |  |
| > 5 | 0.676(0.462-0.989) | 0.044 | 0.782(0.522-1.170) | 0.232 |
| **Number of prescribed doses** |  |  |  |  |
| 0 ~ 20 | Reference |  | Reference |  |
| > 20 | 0.643(0.473-0.874) | 0.005 | 0.774(0.555-1.080) | 0.132 |

Model 1: Crude model.

Model 2 was adjusted for severity of COVID-19, duration of medication treatment, time from hospitalization to initiation of antiviral therapy, and number of prescribed doses.

Abbreviations: HR, hazard ratio; 95% CI, 95% confidence interval; BMI, body mass index.

Table S3 Univariate and multivariate Cox proportional hazards regression analysis of the proportion of ΔCt value for main cohort.

| **Factor** | **Model 1** |  | **Model 2** |  |
| --- | --- | --- | --- | --- |
|  | **Crude HR 95% CI** | **p-**  **value** | **Adjusted HR 95% CI** | **p-**  **value** |
| **Group** |  |  |  |  |
| Naimatevir-Ritonavir | Reference |  | Reference |  |
| Azvudine | 1.346(1.021-1.773) | 0.035 | 1.442(1.084-1.918) | 0.012 |
| **Gender** |  |  |  |  |
| Male | Reference |  |  |  |
| Female | 1.015(0.781-1.319) | 0.910 |  |  |
| **Age** | 0.995(0.980-1.011) | 0.554 |  |  |
| **BMI** | 0.993(0.969-1.018) | 0.600 |  |  |
| **Smoking** |  |  |  |  |
| No | Reference |  |  |  |
| Yes | 0.806(0.597-1.089) | 0.161 |  |  |
| **Drinking** |  |  |  |  |
| No | Reference |  |  |  |
| Yes | 0.815(0.562-1.182) | 0.281 |  |  |
| **Severity of COVID-19** |  |  |  |  |
| Mild-to-moderate | Reference |  | Reference |  |
| Severe | 0.672(0.522-0.866) | 0.002 | 0.757(0.582-0.985) | 0.038 |
| **Comorbidity with diabetes** |  |  |  |  |
| No | Reference |  |  |  |
| Yes | 1.095(0.841-1.424) | 0.500 |  |  |
| **Comorbidity with cardiovascular diseases** |  |  |  |  |
| No | Reference |  |  |  |
| Yes | 1.228(0.921-1.637) | 0.162 |  |  |
| **Comorbidity with hypertension** |  |  |  |  |
| No | Reference |  |  |  |
| Yes | 0.949(0.739-1.219) | 0.682 |  |  |
| **Duration of medication treatment** |  |  |  |  |
| 0 ~ 5 0 | Reference |  | Reference |  |
| 5 ~ 10 | 0.891(0.665-1.193) | 0.437 | 0.824(0.611-1.110) | 0.204 |
| > 10 | 0.594(0.410-0.861) | 0.006 | 0.563(0.385-0.825) | 0.003 |
| **Time from hospitalization to initiation of antiviral therapy** |  |  |  |  |
| ≤ 5 | Reference |  | Reference |  |
| > 5 | 0.710(0.520-0.971) | 0.032 | 0.770(0.554-1.071) | 0.121 |
| **Number of prescribed doses** |  |  |  |  |
| 0 ~ 20 | Reference |  | Reference |  |
| > 20 | 0.766(0.596-0.986) | 0.039 | 0.914(0.696-1.201) | 0.519 |

Model 1: Crude model.

Model 2 was adjusted for severity of COVID-19, duration of medication treatment, time from hospitalization to initiation of antiviral therapy, and number of prescribed doses.

Abbreviations: HR, hazard ratio; 95% CI, 95% confidence interval; BMI, body mass index.
